# 1734. Influenza, SARS-CoV-2, and routine childhood vaccines – trends in vaccine hesitancy in hospitalized children before and during the COVID-19 pandemic

**DOI:** 10.1093/ofid/ofad500.1565

**Published:** 2023-11-27

**Authors:** Marisa Orbea, Anurag Panjabi, Michelle Lopez, Rachel Cunningham, Julie A Boom, C Mary Healy, Claire Bocchini

**Affiliations:** University of Miami Miller School of Medicine, Miami, Florida; Baylor College of Medicine, Houston, Texas; Baylor College of Medicine, Houston, Texas; Texas Children's Hospital, Houston, Texas; Texas Children’s Hospital, Houston, Texas; Baylor College of Medicine, Houston, Texas; Baylor College of Medicine, Houston, Texas

## Abstract

**Background:**

Vaccine hesitancy (VH) is adversely affecting the public health response to the COVID-19 pandemic. Similarly, influenza vaccine uptake is suboptimal.

We monitored trends in VH to influenza, COVID-19, and routine childhood vaccines.

**Methods:**

A repeated cross-sectional survey in English and Spanish of caregiver influenza and COVID-19 knowledge, attitudes, behaviors, and associated VH among hospitalized children 6 mo-18 yrs at a pediatric hospital. We enrolled over 4 seasons (S); ‘19-20 (S1), ‘20-21 (S2), ‘21-22 (S3), ‘22-23 (S4). In S4, we targeted caregivers of children 6 mo-11 yrs. VH was assessed using the Parent Attitudes about Childhood Vaccines (PACV) survey; PACV score ≥ 50 denoted VH.

In S4, a messaging intervention was piloted. Caregivers were randomized to watch 1 of 2 COVID-19 educational videos; half watched a second video featuring a family adversely impacted by COVID-19. All caregivers completed a survey to assess acceptance and efficacy of the video(s) in changing intent to vaccinate their child.

Figure 1.

**Figure:**
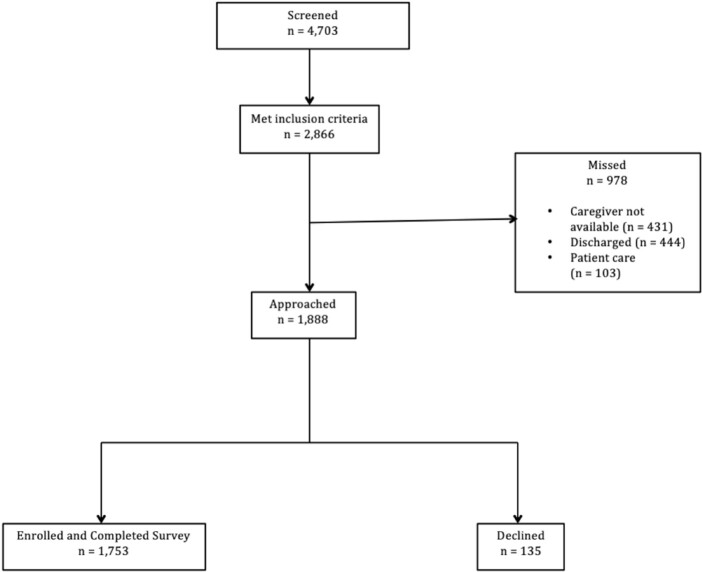

Participant flow chart from 2019-2023

**Results:**

Across all seasons, ≥ 92% of caregivers approached were enrolled. Most (48%) identified as Hispanic/Latino, 35% as White, and 19% as Black. By parental report, 94% of children in S1, 91% in S2, 91% in S3, and 89% in S4 were up-to-date with routine vaccines. Based on PACV score, 13% were VH in S1, 17% in S2, 19% in S3, and 20% in S4 (p=0.14).

During S2-3, fewer caregivers endorsed “flu can be a dangerous infection in children” and “I am scared of my child getting the flu” (p< 0.01). Decreased concern recovered in S4 but did not translate to increased vaccine uptake (Table 1).

Caregivers were less scared of their child getting COVID-19 and more scared of the vaccine in S4. Fewer caregivers in S4 were willing to receive the COVID-19 vaccine; 46% in S2, 54% in S3, and 29% in S4 had/planned to vaccinate their child (Table 2).

Of 800 caregivers, 74% liked the educational videos and 54% thought they were helpful when considering the COVID-19 vaccine for their child. Of 399, 73% liked the family story and 50% thought it was helpful.

Table 1.

**Figure:**
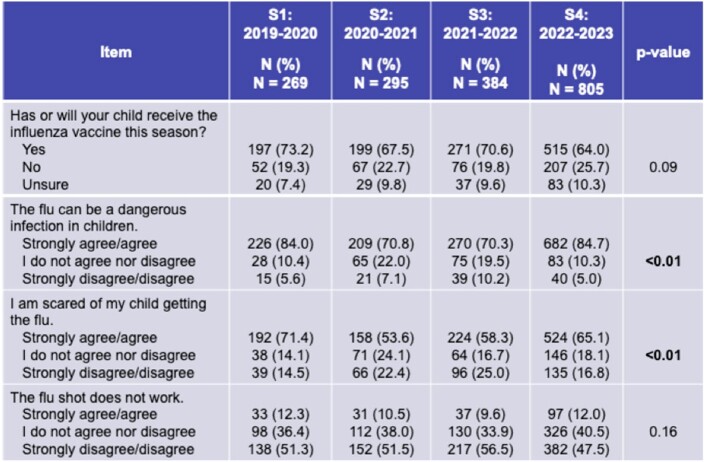

Trends in caregiver attitudes regarding the influenza vaccine

Table 2.

**Figure:**
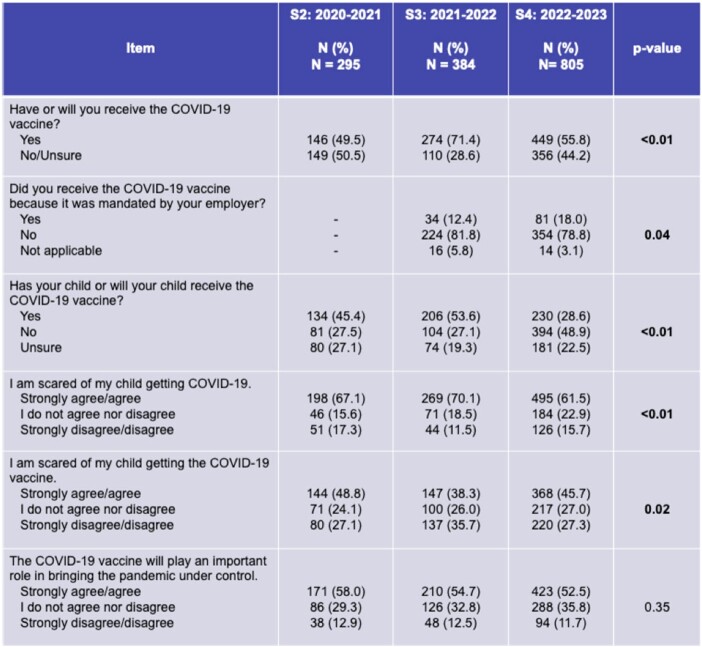

Trends in caregiver attitudes and vaccine acceptance during the COVID-19 pandemic

**Conclusion:**

Parental concern regarding influenza is largely back to pre-pandemic levels but vaccine acceptance has not recovered. Vaccine uptake against COVID-19 remains suboptimal. Educational videos may help caregivers when deciding about the COVID-19 vaccine for their child.

**Disclosures:**

**C.Mary Healy, MD**, Dexcom Inc: Stocks/Bonds|Intuitive: Stocks/Bonds|Quidel Corporation: Stocks/Bonds|Up to Date: Honoraria|Vapotherm: Stocks/Bonds **Claire Bocchini, MD**, Pfizer: Local sub-I for pediatric SARS-CoV-2 vaccine trials

